# Supply chain stability: A study on the enabling effect of state-owned capital

**DOI:** 10.1371/journal.pone.0342691

**Published:** 2026-03-12

**Authors:** Runji Guan, Guangsi Zhang, Feifei Han, Xian Chen

**Affiliations:** 1 School of Business Administration, Xinjiang University of Finance and Economics, Urumqi, China; 2 School of Management, Wenzhou Business College, Wenzhou, China; Peking University, CHINA

## Abstract

Enhancing the resilience and security of industrial and supply chains is a key initiative to drive high-quality development of the real economy, yet the role of state-owned capital participation in this context remains insufficiently understood. This study empirically investigates how state-owned capital involvement affects the supply chain stability of private enterprises, addressing a significant gap in the literature on state-owned capital’s impact on supply chain management. Drawing on data from Chinese A-share-listed private enterprises spanning 2013–2022, this study adopts an empirical research design grounded in empowerment theory to construct models that assess the effect of state-owned capital participation on supply chain stability. Mechanism tests and heterogeneity analyses are conducted to identify mediating pathways and boundary conditions. The results suggest that state-owned capital participation significantly enhances the supply chain stability of private enterprises. State-owned capital influences private enterprises through two mechanisms: resource enabling and optimization of internal governance structures. Heterogeneity analysis further reveals that the positive effect is more pronounced among private enterprises with weaker bank-enterprise relationships, lower product competitiveness, poorer internal control systems, and higher levels of negative media coverage. By clarifying the stabilizing role of state-owned capital participation in enhancing supply chain stability, this study contributes to both supply chain management theory and the impact of state-owned capital on private enterprises. It also provides policy strategies to strengthen industrial and supply chain resilience and security, while offering actionable insights into targeted policy interventions tailored to different types of private enterprises.

## 1. Introduction

The current international political, economic, and trade landscape is increasingly volatile, leading to recurrent disruptions in global supply chains, manifested as “congestion,” “bottlenecks,” and “disruptions.” In this environment, enhancing the resilience and security level of global industrial and supply chains has become a pressing priority. Governments worldwide are adjusting their policies and strategies to enhance supply chain resilience, aiming to shape safer and more sustainable supply chains. On February 24, 2021, the US government launched a supply chain “absolute security” initiative from an “America first” perspective, reshaping its supply chain architecture through domestic investment and international realignment. Similarly, on July 18, 2024, the Third Plenary Session of the 20th Central Committee of the Communist Party of China explicitly emphasized the need to enhance the resilience and security of industrial and supply chains. In this context, supply chain stability has emerged as a key determinant of overall supply chain resilience [1,2]. Supply chain stability refers to the expectation of sustained and reliable cooperation between enterprises. It is characterized by long-term relationships with a diverse set of upstream suppliers and downstream customers. Specifically, stable upstream linkages can ensure consistent access of raw material supply; correspondingly, stable downstream connections can facilitate predictable finished product inventory turnover and market access. Conversely, instability in these relationships not only forfeit all the advantages brought by relational capital but also incurs substantial search and switching costs, thereby increasing the vulnerability of supply chain disruptions [3]. A notable example is OFILM, a company specializing in touchscreen technology, which suffered a 93% decline in net profit in 2021 due to Apple Inc.’s termination of their partnership. Given the current domestic and international macroeconomic situation, effectively enhancing the stability of enterprise supply chains has become an important and urgent practical issue for both academia and the business community.

As of May 2024, private enterprises accounted for 96.4% of the total number of enterprises in China, positioning them as central drivers of national modernization and essential pillars for sustaining high-quality economic growth. Therefore, ensuring the stability of private enterprise supply chains is vital to strengthening the resilience and security of China’s industrial and supply chains. However, compared to state-owned enterprises (SOEs), most private enterprises less preferred partners for both upstream suppliers and downstream customers [4]. This results in greater volatility in their supply chain relationships. One key reason lies in the actual controllers of state-owned enterprises: their ultimate controllers are all-level governments, endowing them with higher perceived credibility, policy support, and long-term viability. These enterprises inherently benefit from the endorsement of national credit and are more likely to obtain various government subsidies and policy incentives [5]. This greatly reduces suppliers’ and customers’ concerns about the debt repayment ability and sustainable supply ability of enterprises. As a result, it is more favorable for suppliers and customers to form long-term and stable business relationships with state-owned enterprises.

Based on the above analysis, state-owned capital participation can theoretically enhance the stability of private enterprises’ supply chains. However, existing studies on state-owned capital participation have predominantly focused on its effects in on private enterprises’ financing constraints [6], patent output [7], promoting innovation investment [8], and improving information disclosure quality [9], while largely overlooking its implications for supply chain stability. Given this gap, this study raises a critical question: Can private enterprises leverage national credit endorsement through state-owned capital participation to positively impact their supply chain stability?

In practice, China’s reverse mixed-ownership reform provides a natural setting for examining how private firms gain institutional credibility via state capital involvement. In 2015, the State Council of China issued the “Guiding Opinions on Deepening the Reform of State-owned Enterprises,” explicitly encouraging state-owned enterprises to take equity stakes in non-state-owned enterprises through various means. Under this policy impetus, reverse mixed-ownership reform projects, where state capital enters private enterprises as minority shareholders, have been gradually implemented in depth. Existing research has shown that state capital exerts both resource and governance effects [10], which significantly shape the resource endowments and governance levels of private enterprises, thereby altering their business behavior. For example, state capital participation helps private enterprises mitigate stock price crashes [11], improve ESG performance [12], and reduce management cost [13]. Nevertheless, previous literature on reverse mixed-ownership reform has mainly centered on the internal characteristics of private enterprises [10,14,15], with little research examining the supply chain management level. To date, no systematic study has investigated the impact of state capital participation on the supply chain stability of private enterprises. Against the broader strategic imperative of strengthening industrial and supply chain resilience, this study addresses three key questions: Does supply chain stability in private enterprises improve following state capital entry? If so, what is the underlying mechanism? And do these effects vary across different scenarios? To answer these questions, this study employs empowerment theory, selects data from A-share listed private enterprises from 2013 to 2022 to empirically examine the causal relationship and mechanism linking state capital participation to supply chain stability.

The contributions of this study are threefold. First, based on the empowerment theory, this study constructs a theoretical analysis framework that examines how state capital participation enhances the stability of private enterprises’ supply chains, systematically extending research on state capital participation to the field of supply chain management. It provides a theoretical framework for private enterprises in developing countries to introduce state capital and enhance supply chain stability in the face of fluctuations. Second, it identifies and tests two mediating pathways: resource enabling (alleviating resource constraints) and governance improvement (mitigating principal-agent conflicts), thereby clarifying the channels through which state capital participation influences private enterprises and enriching the existing transmission paths of the impact of state capital participation on private enterprises. Third, by conducting fine-grained subgroup analyses based on whether there is a bank-enterprise relationship, product competitiveness, internal control quality, and the frequency of negative media reports, this study reveals the heterogeneous impact of state capital participation on the supply chain stability of private enterprises across different groups, offering targeted policy recommendations for optimizing state capital allocation.

## 2. Literature review

### 2.1. Research on state-owned capital participation

Mixed-ownership reform, as a strategic initiative for deep-level adjustment of the national economic structure and improvement of the socialist market economic system, has attracted extensive attention in academia. Early studies primarily focused on the forward mixed-ownership reform, where state-owned enterprises introduced private equity, investigating the impact of non-state shareholders on aspects such as internal control quality [16], executive compensation stickiness [17], and the economic performance of state-owned enterprises.

In recent years, with the deepening of mixed-ownership reform, reverse mixed-ownership has emerged as a significant research focus, representing another key model of ownership integration. Scholars have mainly employed empirical research methods to examine the economic consequences of state-owned capital participation, which can be broadly categorized into two dimensions: political connections and corporate governance. At the political connection level, the presence of state-owned capital establishes a direct institutional link between private firms and the government, allowing such participation to be viewed as a unique form of political affiliation [18]. By leveraging the political attributes of state-owned capital [19], private enterprises can significantly alleviate the resource constraints caused by ownership-related factors [6,20,21], thereby exerting positive effects on corporate innovation investment [22], digital transformation [7], and risk-taking [20]. However, this benefit may come at a cost, such as increased labor cost stickiness [21]. On the other hand, some studies also point out that while private firms gain access to valuable resources, they may also face pressures from government intervention, including absorbing excess employment [23] and engaging in more frequent charitable donations [24]. At the corporate governance level, state-owned capital can function as an active monitoring entity, helping mitigate the dual agency problem of private enterprises [10,25]. This not only improves the quality of corporate disclosure [26] and reduces the likelihood of misconduct [27], but also significantly curbs corporate financialization [25]. In addition, state capital involvement can further stimulate managerial innovation, thereby contributing positively to firms’ financial performance [26].

### 2.2. Research on supply chain stability

Another stream of literature closely related to this study focuses on supply chain stability. Prior studies have examined the economic implications of supply chain stability. From the perspective of a company’s own development, maintaining long-term and stable cooperative relationships with suppliers and customers facilitates greater access to trade credit financing [28], improve green innovation [29], and improve investment efficiency [30], all of which support enterprise innovation and stabilize production expectations [31]. Meanwhile, supply chain stability can also send positive signals externally, alleviating information asymmetry with other stakeholders, thereby increasing bank borrowings [32] and improving the enterprise’s information environment [33]. Conceptually, supply chain stability refers to the stability of the supply chain to maintain consistent operations amid external disturbances, serving as a core component of supply chain resilience [34].

Given the complex and volatile geopolitical and economic environment, supply chain stability is crucial for enterprises to cultivate competitive advantages. Currently, based on data disclosed by Chinese listed companies, the purchase and sale amounts with the top five suppliers and top five customers account for 30–40% of the annual operating revenue for most enterprises, indicating a heavy dependence on major suppliers and customers [35]. Therefore, exploring how to maintain supply chain stability has important practical significance. Baiman and Rajan (2022) argued that reducing information asymmetry between suppliers and customers significantly strengthens supply chain stability [36]. Bauer et al. (2018) conducted research on the relationship between a company’s internal control level and supply chain stability, finding that a robust internal control level can significantly reduce the occurrence of supply chain disruptions, thereby enhancing supply chain stability [37]. Chen et al. (2024) found that ESG decoupling undermines customer stability by damaging corporate reputation and exacerbating information asymmetry [38]. Jin (2025) noted that the heightened concentration of expert power within firms can deteriorate the quality of corporate disclosures, thereby indirectly undermining supply chain stability [39].

### 2.3. Research gap

Existing literature on supply chain stability has mainly explored influencing factors such as alleviating information asymmetry, improving internal corporate governance, enhancing ESG performance, and the involvement of venture capital in equity structures. Meanwhile, research on economic effects of state-owned capital participation, prior studies have largely focused on its impact on private enterprises’ innovation investment and digital transformation. However, in the today’s economic environment, competition among firms has increasingly shifted toward competition between supply chains. Supply chain stability, as a core indicator of a firm’s supply chain management capability, is crucial for private enterprises to withstand external risks and achieve sustainable development. Nevertheless, existing research has largely overlooked state-owned capital’s influence into the realm of corporate supply chain management. This gap may hinder a comprehensive understanding of the driving mechanisms behind supply chain stability. Therefore, it is imperative to investigate how state-owned capital participation affects the supply chain stability of private enterprises.

Considering the above research gap, this study innovatively incorporates state-owned capital participation and the supply chain stability of private enterprises into a unified research framework. It combines empowerment theory with empirical research methods. The study systematically investigates and tests the impact of state-owned capital involvement on the supply chain stability of private enterprises, as well as the underlying mechanisms. The goal is to provide a theoretical basis and decision-making reference for optimizing supply chain management through strategic equity arrangements. It also offers new empirical evidence on the interaction between state-owned capital and supply chain governance. This contributes to the high-quality development of the private economy and enhances industrial and supply chain resilience.

## 3. Theoretical analysis and hypothesis formation

In recent years, the empowerment theory has gained significant importance in psychology, sociology, and organizational management [40,41]. At its core, empowerment involves enhancing the resource endowments and integration capabilities of individuals or organizations through authorization and capacity building. With the ongoing advancement of China’s reverse mixed-ownership reform, the concept of state-owned capital empowerment has emerged. Through equity participation, it alters the resource acquisition capabilities and ownership structure of private enterprises through equity participation, thereby empowering them with resources and enhancing their governance structure. Considering this, this study argues that state-owned capital participation can influence the stability of a company’s supply chain through two paths: resource empowerment effect and governance structure empowerment.

### 3.1. Resource empowerment – alleviating resource constraints

Grounded in the resource-based view (RBV), the resource empowerment provided by state-owned capital to private enterprises helps alleviate their resource constraints, thereby offering critical support for supply chain stability. The RBV posits that the heterogeneous resources possessed by firms are the core source of their competitive advantage [42]. State-owned capital participation addresses resource deficiencies in private firms by injecting scarce and valuable resources, forming the theoretical foundation of the resource empowerment mechanism. Specifically, the resource empowerment effect manifests primarily in two dimensions: financial resources and political resources, both of which are aligned with the RBV principles of “scarcity” and “value.” In terms of financial resources, state-owned enterprises, with their high credit ratings and stable funding sources, possess high-quality financial resources that are difficult for private firms to access. Once state-owned capital becomes a shareholder, private enterprises can leverage these advantages to secure more favorable loan terms, including more favorable loan interest rates and longer repayment periods when applying for bank loans or issuing bonds [43]. From the perspective of resource signaling, state-owned capital participation signals to the market that the enterprise’s quality has been endorsed by authoritative entities [24]. Backed by the credibility of state-owned capital, this signal reduces information asymmetry for external investors, enabling private enterprises to attract more external financing. For instance, after Guozhen Environmental introduced China Energy Conservation and Environmental Protection Group as a state-owned shareholder, it secured an additional credit line of 3.7 billion yuan through the group’s platform resources, significantly easing financing constraints, a clear demonstration of financial resource empowerment. In terms of political resources, the integration of state-owned and private capital fosters a “symbiotic relationship” at the institutional level, allowing private enterprises to benefit from the political connections of their state-owned shareholders. Leveraging their close ties with government agencies, state-owned shareholders can assist private firms in securing policy support, such as reduced thresholds for government subsidies [44], obtaining tax incentives, and gaining market access in regulated industries [45]. These political resources are scarce and difficult to imitate, aligning with the RBV’s definition of “strategic resources” and providing private enterprises with unique competitive advantages.

The enhancement of resource acquisition capabilities resulting from the above-mentioned resource empowerment has a direct and tangible impact on supply chain stability. In the resource-based view, “resource application capability” acts as the critical mediating mechanism. In supplier relationship management, private enterprises with ample financial resources and stable operations can offer suppliers reliable credit guarantees and substantial order commitments [46]. This cooperation model is grounded in resource advantages and fosters long-term trust from suppliers. Consequently, suppliers prioritize these firms during periods of resource scarcity. For example, stable cash flow enables timely payment for goods and services, preventing disruptions in cooperation caused by suppliers’ liquidity constraints and thereby maintaining supply chain continuity. In terms of technological innovation investment, abundant resource reserves allow private enterprises to increase spending on supply chain management technologies [47]. These may include adopting Internet of Things (IoT) solutions and advanced supply chain management software [48]. Such technological investments enhance supply chain transparency and early-warning capabilities for potential risks. By tracking real-time data on raw material transportation, inventory levels, and other key indicators, firms can identify emerging supply disruption risks and proactively adjust strategies [49]. This resource-based technological empowerment directly strengthens the supply chain’s resilience in coping with uncertainties.

### 3.2. Internal governance structure empowerment – Mitigating principal-agent conflicts

Agency theory suggests that state-owned capital empowers the governance structures of private enterprises. This empowerment helps reduce principal, agent conflicts by improving governance mechanisms, thereby strengthening supply chain stability. When ownership is separated from management, conflicts can arise due to different interests and information gaps. A sound governance structure helps balance the relationship between shareholders and management [50], supporting the role of state-owned capital in this process.

Specifically, governance structure empowerment operates mainly through three dimensions: First, governance structure optimization. State-owned enterprises typically possess mature governance systems and standardized management practices [51]. Their participation in private enterprises facilitates the transfer of institutional experience, assisting in clarifying the division of rights and responsibilities among shareholders, boards of directors, and management teams. From the perspective of agency theory, this clear allocation of authority and responsibility reduces the room for agents to overstep their bounds or shirk duties, directly lowering the goal divergence between shareholders and management and easing the core principle of agent conflicts. Second, an improvement in information transparency. Information asymmetry is a primary driver of principal-agent conflicts [52]. Following state-owned capital participation, SOE oversight prompts private enterprises to improve the quality and transparency of their information disclosure. By reducing shareholders’ “information disadvantage” regarding managerial actions, this shift curbs managers’ moral hazard and opportunistic behavior [9], further weakening agency tensions. Third, refinement of compensation and incentive mechanisms. State-owned capital involvement can encourage the adoption of scientific incentive and accountability systems in private enterprises [53]. On one hand, linking compensation mechanisms to shareholder returns aligns the managerial incentives with those of shareholders; on the other hand, strengthened oversight and performance evaluation mechanisms impose behavioral constraints on management [54]. Together, these dual mechanisms address principal-agent issues from both “interest alignment” and “behavioral control” perspectives, effectively reducing potential conflicts.

The mitigation of principal-agent conflicts through governance structure empowerment is directly linked to supply chain stability via three transmission channels: First, in the trust and cooperation dimension, standardized internal governance and reduced agency conflicts signal to supply chain partners that the enterprise operates in a “stable and risk-controlled” manner [55]. This signal, derived from the predictability due to governance optimization, directly enhances the trust of suppliers, distributors, and other partners. Since trust underpins long-term supply chain collaboration, its reinforcement significantly reduces the likelihood of opportunistic behavior and promotes deeper, more stable partnerships. Second, in the information sharing and strategic coordination dimension, the mitigation of agency conflicts removes barriers to internal information transmission [56], enabling the firm to share critical data such as demand forecasts and inventory levels more effectively with supply chain partners. This seamless flow of information, resulting from improved internal coordination efficiency due to governance optimization, strengthens strategic alignment among supply chain members, reduces supply disruptions caused by information delays or inaccuracies, and thereby improves overall stability [57]. Third, in the dimension of internal operations and external interactions, reduced agency conflicts directly optimize internal business processes [58], making procurement, production, and logistics decisions more efficient. This internal efficiency extends into supply chain interactions, thus reducing supply chain volatility stemming from poor internal coordination.

In summary, this study proposes the following research hypotheses:

H1: State-owned capital participation enhances the supply chain stability of private enterprises.
H1a: State-owned capital participation can enhance the supply chain stability of private enterprises by alleviating resource constraints.
H1b: State-owned capital participation can enhance the supply chain stability of private enterprises by alleviating principal-agent conflicts.

## 4. Research design

### 4.1. Data source

In 2013, the Third Plenary Session of the 18th Central Committee of the Communist Party of China proposed encouraging mixed-ownership enterprises with non-state capital as the controlling shareholder, marking the beginning of the reverse mixed-ownership reform initiative. Accordingly, this study examines A-share listed private enterprises from 2013 to 2022. Based on prior research, data were processed as follows: (1) Financial firms were excluded due to major differences in business models, regulatory environments, and supply chain structures. (2) Companies designated as ST, *ST, or those with severe data deficiencies were removed to minimize bias from financial distress or incomplete reporting. (3) Firms with state-owned shareholders holding more than 50% were excluded to focus specifically on non-controlling state-owned participation. (4) Continuous variables were winsorized at the 1% level to mitigate the influence of outliers. After these adjustments, the sample comprised 8,873 observations from 1,392 private listed companies. Unless otherwise specified, all variables used in subsequent analyses were obtained from the CSMAR and Wind databases.

### 4.2. Variable definition

#### 4.2.1. Dependent variable: Supply chain stability.

With reference to existing research [28], this study constructs a three-dimensional variable for supply chain stability: (1) Supplier Relationship Stability (SRS): calculated as the number of the top five suppliers from the previous year that remain among the top five suppliers in the current year, divided by 5; (2) Customer Relationship Stability (CRS): Measured by the number of the top five customers from the previous year that remain in the list of the top five customers in the current year, divided by 5; (3) Overall Supply Chain Stability (Stability): Measured by the average of SRS and CRS. All three-dimensional variables are all positive indicators, meaning the higher the value, the stronger the stability of the supply chain. In this paper, Stability is used as the main explanatory variable in the benchmark model regression, while SRS and CRS are used as supplementary indicators for robustness testing to ensure the universality and reliability of the research conclusions.

#### 4.2.2. Core explanatory variable: State-owned capital participation.

With reference to existing research [43], this study employs three proxy variables to capture state-owned capital participation: (1) State-owned Shareholding Proportion (State1), measured as the sum of the shareholding proportions of state-owned shareholders among the top ten shareholders of private enterprises, that is the sum of the shareholding ratios of all state-owned shareholders among the top ten shareholders divided by the sum of the shareholding ratios of the top ten shareholders; (2) Degree of Equity Balance (State2), measured by the ratio of state-owned shareholders’ shareholdings to non-state-owned shareholders’ shareholdings among the top ten shareholders; (3) Presence of Major State-owned Shareholder (State3), assigned a value of 1 if the largest state-owned shareholder holds more than 10% of shares, and 0 otherwise. All three indicators are positively associated with State 1 participating in the benchmark regression, while States 2 and 3 are employed in robustness tests.

#### 4.2.3. Control variables.

To address potential omitted variables, the model includes these control variables: (1) General corporate characteristics: Firm Size (Size), measured by the natural logarithm of year-end total assets; and Firm Age (Age), calculated as the natural logarithm of the number of years since establishment. (2) Financial characteristics: Asset-Liability Ratio (Lev), defined as ratio of total liabilities to total assets; and Profitability (ROA), defined as return on total assets. (3) Corporate governance: Largest Shareholder Holding (Top1), expressed as the percentage shareholding of the largest shareholder; Board Size (Board), measured by the natural logarithm of the number of board members; Duality of CEO and Chairman (Dual), set to 1 if the general manager and chairman are the same, otherwise 0; Audit Firm Size (Big4), set to 1 if audited by a Big Four firm, otherwise 0. (4) Year (Year) and firm (Firm) fixed effects control for time- and firm-level influences.

### 4.3. Model construction

To examine the impact of state-owned capital participation on the supply chain stability of private enterprises, this study constructs a two-way fixed effects model (1):


$${Stability}_{i,t}=\alpha_{\text{0}}+\alpha_{\text{1}}{State\text{1}}_{i,t}+\alpha_{\text{2}}Controls+Year+Firm+\varepsilon_{i,t}$$ (1)

Where, the subscripts *i* and *t* denote the enterprise and the year respectively; ${Stability}_{i,t}$ represents supply chain stability; ${State\text{1}}_{i,t}$ represents the degree of state-owned capital participation in private enterprises; Controls represent all control variables; Year and Firm represent year fixed effects and enterprise fixed effects respectively; $\varepsilon_{i,t}$ represents the random disturbance term.

## 5. Empirical analysis

### 5.1. Descriptive statistics

The descriptive statistics results for the main variables in this study are presented in Table 1. The variable Stability ranges from 0 to 1, indicating significant differences in supply chain stability among different private enterprises. Meanwhile, the mean value of Stability is 0.583, suggesting that at least two of the top five suppliers or customers of sample firms experienced relationship changes within the given year. In addition, the mean value of the core explanatory variable, State1,is only 0.018 with a standard deviation of 0.035, indicating low overall levels of state-owned capital participation among Chinese private enterprises, and most of them do not have state shareholders among their top ten shareholders. Higher values of State1 signals greater state-owned capital involvement. The remaining variables all fall within a reasonable range and consistent with patterns observed in existing literature.

**Table 1:** Descriptive Statistics Results of Main Variables.

*VARIABLES*	Obs	Mean	Std. Dev.	Min	Median	Max
*Stability*	8873	0.583	0.351	0	0.6	1
*State1*	8873	0.018	0.035	0	0	0.205
*Size*	8873	22.011	1.071	19.976	21.902	25.348
*Lev*	8873	0.387	0.191	0.057	0.377	0.854
*ROA*	8873	0.037	0.073	−0.253	0.039	0.22
*Board*	8873	2.078	0.186	1.609	2.197	2.485
*Dual*	8873	0.373	0.484	0	0	1
*Top1*	8873	30.152	13.548	8.544	28.229	68.099
*Age*	8873	2.063	0.751	0	2.197	3.296
*Big4*	8873	0.033	0.178	0	0	1

### 5.2. Correlation analysis

This study employs the Pearson correlation test to investigate the relationship between variables. As shown in Table 2, the correlation coefficient between State1 and Stability is 0.135, which is significantly positive at the 1% level, indicating a robust positive association between state capital participation and supply chain stability in private enterprises. This provides preliminary support for Hypothesis 1.

**Table 2:** Results of Correlation Analysis.

VARIABLES	*Stability*	*State1*	*Size*	*Lev*	*ROA*	*Board*	*Dual*	*Top1*	*Age*	*Big4*
*Stability*	1									
*State1*	0.135***	1								
*Size*	0.214***	0.135***	1							
*Lev*	0.083***	0.077***	0.450***	1						
*ROA*	−0.018*	−0.003	0.060***	−0.329***	1					
*Board*	0.034***	0.102***	0.158***	0.066***	0.058***	1				
*Dual*	−0.016	−0.013	−0.061***	−0.038***	0.004	−0.111***	1			
*Top1*	−0.021*	−0.104***	0.037***	−0.028***	0.197***	−0.028***	0.032***	1		
*Age*	0.314***	0.156***	0.408***	0.299***	0.193***	0.053***	−0.103***	−0.205***	1	
*Big4*	0.040***	0.065***	0.224***	0.049***	0.034***	0.026**	−0.023**	0.046***	0.020*	1

### 5.3. Benchmark regression

Table 3 reports the benchmark regression results of the impact of state-owned capital participation on the supply chain stability of private enterprises. To enhance the reliability of the research conclusions, a stepwise modeling strategy is adopted, gradually incorporating control variables and fixed effects. As shown in Table 3, across all specifications, the coefficient on State1 remains positive and statistically significant at the 1% level, regardless of model adjustments. Meanwhile, as control over the model tightens, the R-squared value increases. With enhanced control, the model’s explanatory power gradually improves. From an economic perspective, taking the fourth example where all control variables and fixed effects are included, for a one-standard-deviation increase of state-owned capital participation, the supply chain stability of private enterprises increases by 0.020 (0.5713 × 0.035) units, which is a 3.42% (0.020/ 0.583) increase compared to the mean. Therefore, from either an economic or statistical perspective, state-owned capital participation has a statistically and economically meaningful positive effect on supply chain stability in private enterprises, offering initial validation of Hypothesis 1. Hypothesis 1 of this paper is initially verified.

**Table 3:** Benchmark Regression Results.

VARIABLES	(1)	(2)	(3)	(4)
	*Stability*	*Stability*	*Stability*	*Stability*
*State1*	1.3456^***^	0.8387^***^	0.8341^***^	0.5713^***^
	(0.1047)	(0.1017)	(0.1016)	(0.1641)
*Size*		0.0361^***^	0.0386^***^	0.0134
		(0.0042)	(0.0043)	(0.0084)
*Age*		0.1326^***^	0.1346^***^	−0.0825^***^
		(0.0054)	(0.0055)	(0.0188)
*Lev*		−0.1015^***^	−0.1068^***^	−0.1421^***^
		(0.0224)	(0.0225)	(0.0333)
*Roa*		0.0191	−0.0026	−0.1947^***^
		(0.0540)	(0.0542)	(0.0552)
*Board*		−0.0021	−0.0067	−0.0066
		(0.0193)	(0.0194)	(0.0304)
*Dual*		0.0126^*^	0.0134^*^	−0.0149
		(0.0073)	(0.0073)	(0.0096)
*Top1*		0.0010^***^	0.0009^***^	−0.0006
		(0.0003)	(0.0003)	(0.0006)
*Big4*		0.0108	0.0089	−0.0133
		(0.0204)	(0.0203)	(0.0332)
Year	NO	NO	YES	YES
Firm	NO	NO	NO	YES
Constant	0.5594^***^	−0.4927^***^	−0.5369^***^	0.3972^**^
	(0.0041)	(0.0893)	(0.0898)	(0.1799)
*N*	8873	8873	8873	8873
*R* ^2^	0.0183	0.1185	0.1231	0.6105

Note: The numbers in parentheses are robust standard errors clustered at the firm level. ***, **, * represent significance at the 1%, 5%, and 10% levels, respectively.

### 5.4. Robustness test

#### 5.4.1. Instrumental variable method.

Endogeneity arising from potential reverse causality poses a critical challenge. State capital participation can enhance the stability of private enterprise supply chains, while private enterprises with more stable supply chains might also attract greater state investment. The instrumental variable (IV) approach introduces instruments that satisfy two core conditions. (i) strong relevance: they must be highly correlated with the endogenous regressor (State1); and (ii) exogeneity: they must influence the outcome (Stability) only through the regressor and be uncorrelated with the error term or any casual pathway. This process filters out the portion of the explanatory variable influenced by the dependent variable. It retains only its exogenous variation, enables the regression to isolate the causal effect and mitigate bias arising from reverse causality. Therefore, to mitigate potential endogeneity issues, this study conducts an instrumental variable (IV) test. Firstly, following established research practices [11,59], we adopt the industry-year average proportion of state capital participation as an instrumental variable for state capital participation (IV). The reasons are as follows: Firstly, the state capital tends to support key national industries. Therefore, the average level of state capital participation in the industry in which a private enterprise operates is correlated with the level of state capital participation that the enterprise itself can obtain, satisfying the relevance requirement. Secondly, the average proportion of state capital participation in the industry for that year is unlikely to have a direct impact on the stability of the private enterprise’s supply chain, meeting the exclusion restriction. The first-stage regression results, reported in column (1) of Table 4, show a significantly positive coefficient on the IV, confirming a strong association between the instrument and the endogenous regressor. Furthermore, the first-stage F-values exceed the empirical threshold of 10, indicating the reasonableness of the instrumental variable selection. In the second-stage, regression results presented in column (2) of Table 4. In the second stage, presented in column (2) of Table 4, the results show that the regression coefficient of State1 remains significantly positive, confirming the robustness of the baseline regression results.

**Table 4:** Robustness Tests: Instrumental Variable Method, Matching Method, Difference-in-Differences (DID) Method.

VARIABLES	IV		PSM EBM		DID
	(1)	(2)	(3)	(4)	(5)
	*State1*	*Stability*	*Stability*	*Stability*	*Stability*
*State1*		2.5775^***^	0.6543^***^	0.5275^***^	
		(0.9421)	(0.1794)	(0.1572)	
*IV*	0.5105^***^				
	(0.0344)				
Wald F statistic	219.984				
*Treat* × *Post*					0.0494^***^
					(0.0124)
Controls	YES	YES	YES	YES	YES
Year & Firm	YES	YES	YES	YES	YES
Constant	0.0233^*^	0.3122^*^	0.1899	0.3116	0.4346^**^
	(0.0131)	(0.1860)	(0.2217)	(0.2145)	(0.1797)
*N*	8873	8873	6606	8873	8873
*R* ^2^	0.7980	0.0247	0.6049	0.5958	0.6108

#### 5.4.2. Matching method.

Given that many private enterprises in the sample have not received state-owned capital participation, there exists a potential for sample selection bias. The propensity score matching (PSM) method compresses multiple observable characteristics into a single propensity score and selects control group samples with similar characteristics to the treatment group based on this score. This ensures that the matched groups are balanced on observable variables, thereby simulating a counterfactual framework akin to a randomized experiment. It effectively eliminates bias caused by non-random sample selection and enhances the reliability of the estimated results. The entropy balancing method assigns specific weights to control group samples based on pre-specified moment constraints, ensuring that the weighted control group perfectly matches the treatment group in terms of the distribution of observable characteristics. This approach eliminates selection bias arising from differences in characteristic distributions between the two groups. To reduce bias arising from sample selection bias, two matching methods are used: Propensity Score Matching (PSM) and Entropy Balancing Method (EBM). These methods help ensure that private enterprises, whether with or without state-owned capital, share similar basic characteristics. Both methods use all control variables from Model (1) as covariates, with state-owned capital participation serving as the treatment indicator. The study conducts 1:1 nearest neighbor matching and EBM matching. After matching, the benchmark regression model is used again. The regression results for both methods are shown in columns (3) and (4) of Table 4. After matching, the estimated coefficients for State1 are both positive and significant at the 1% level. This suggests that the main findings are robust to corrections for sample selection bias due to observable differences.

#### 5.4.3. Difference-in-differences (DID) method.

To further mitigate the interference caused by reverse causality and sample selection bias in the research, a multi-period difference-in-differences (DID) framework is implemented to reassess the causal relationship between state-owned capital participation and increased supply chain stability of private enterprises. Specifically, referring to the approach of existing research [11], state capital entry is treated as an exogenous policy-like shock, the following DID model is specified:


$${Stability}_{it}=\beta_{\text{0}}+\beta_{\text{1}}{{Treat}_{i}\times Post}_{it}+\beta_{\text{2}}Controls+Year+Firm+\varepsilon_{it}$$ (2)

Where Treat is a grouping dummy variable. If a private enterprise has received state-owned capital participation during the sample period, it is assigned to the treatment group (Treat = 1); otherwise, it is assigned to the control group (Treat = 0). Post is a time dummy variable, that equals 1 in the year of first exposure to state capital and all subsequent years, and 0 in earlier years. The definitions of the remaining variables are consistent with the benchmark regression model. The regression results are shown in column (5) of Table 4. The results indicate that the regression coefficient of Treat × Post is significantly positive at the 1% level, again suggesting that state-owned capital participation has a significant positive impact on enhancing the supply chain stability of private enterprises.

Furthermore, this study employs an event study approach to construct Model (3), aiming to examine the dynamic effects of state-owned capital participation on supply chain stability:


$${Stability}_{it}=\gamma_{\text{0}}+{\Sigma \gamma }_{m}{{Treat}_{i}\times Post(n)}_{it}+\gamma_{\text{2}}Controls+Year+Firm+\varepsilon_{it}$$ (3)

**Fig 1:**
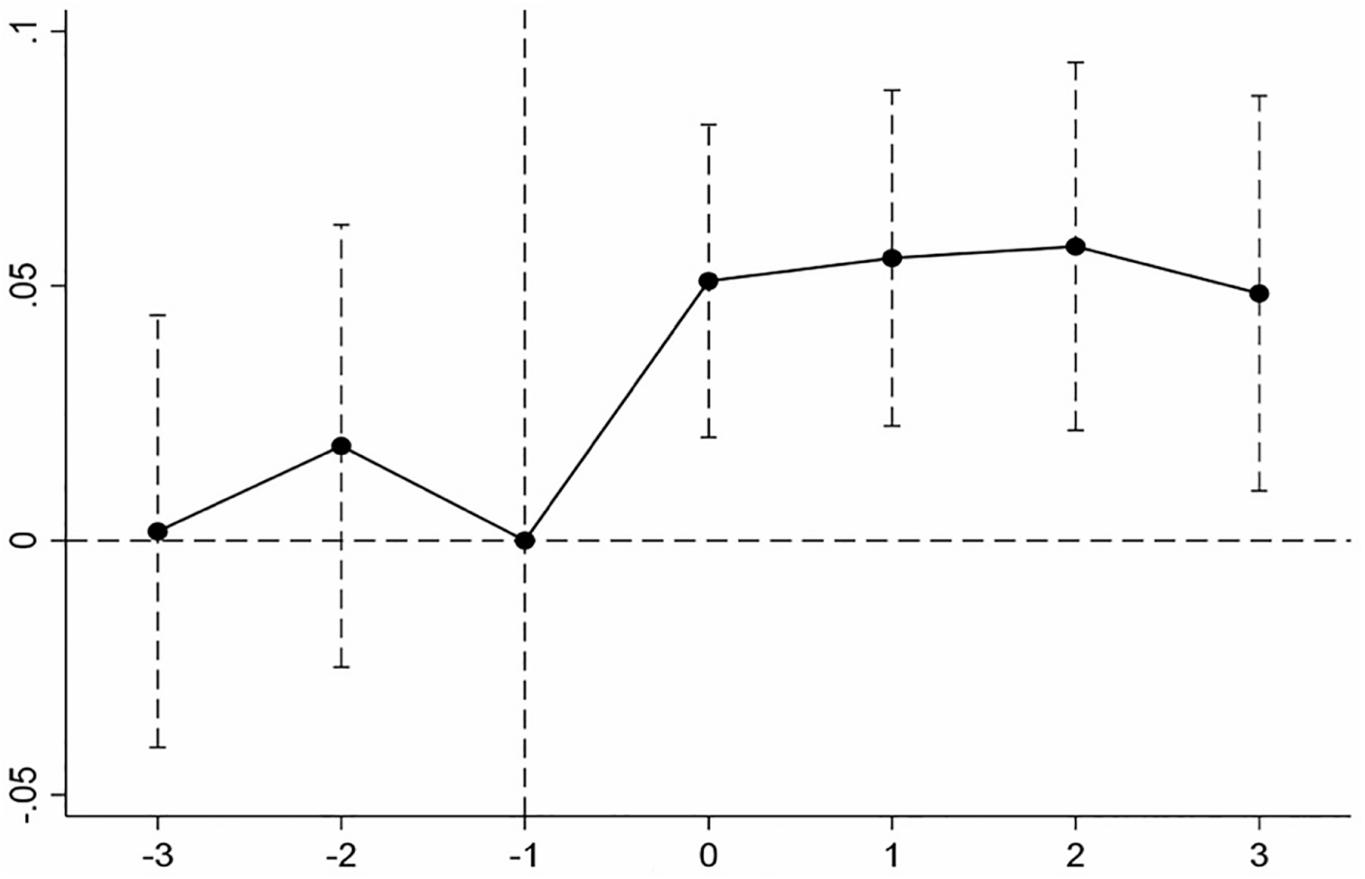
Results of the Parallel Trends Test.

Where Post (n) represents the nth period before and after the first state-owned capital participation event for both the treatment and control groups. In this study, the period immediately preceding the event is set as the reference (base) period to test the parallel trends assumption, and the results are shown in Fig 1. Before state-owned capital participation, there is no significant difference in supply chain stability between the two groups of private enterprises, satisfying the parallel trends assumption. However, following state-owned capital participation, the treatment group exhibits a significant and sustained increase in supply chain stability relative to the control group, indicating a lasting positive impact of state ownership involvement.

#### 5.4.4. Alternative variable specifications.

To further verify that state-owned capital participation can increase the stability of private enterprises’ supply chains, alternative measures of both the key explanatory variable and the dependent variable are employed. First, the equity balance ratio between state-owned shareholders and non-state-owned shareholders among the top ten shareholders (State2) and whether the largest state-owned shareholder holds more than 10% of the shares (State3) are used to re-characterize the measure of state-owned capital participation. The regression results are shown in columns (1) and (2) of Table 5. The results indicate that the regression coefficients of both State2 and State3 are significantly positive. Second, in the previous sections, the average of supplier relationship stability and customer relationship stability was used to measure supply chain stability. Next, this study conducts separate regressions on supplier stability (SRS) and customer stability (CRS). The regression results in columns (3) and (4) of Table 5 show that the regression coefficients for State1 are 0.5310 and 0.6116, both of which are significantly positive at the 1% level, indicating that state-owned capital participation positively promotes both supplier and customer stability. Moreover, the regression coefficient of CRS is greater than that of SRS, suggesting that state-owned capital participation has a relatively stronger effect on customer-side relationships for private enterprises. These results indicate that the main conclusions remain robust under alternative model specifications.

**Table 5:** Other Robustness Tests: Alternative Variable Specifications, Other Influencing Factors.

VARIABLES	(1)	(2)	(3)	(4)	(5)	(6)
	*Stability*	*Stability*	*SRS*	*CRS*	*Stability*	*Stability*
*State1*			0.5310^***^	0.6116^***^	0.4055^**^	0.8006^***^
			(0.1832)	(0.1845)	(0.1719)	(0.2320)
*State2*	0.2068^***^					
	(0.0591)					
*State3*		0.0531^**^				
		(0.0269)				
Controls	YES	YES	YES	YES	YES	YES
Year & Firm	YES	YES	YES	YES	YES	YES
Year × Ind	NO	NO	NO	NO	YES	NO
Constant	0.3777^**^	0.4089^**^	0.4096^**^	0.3849^*^	0.9826^***^	1.0259^***^
	(0.1802)	(0.1800)	(0.2008)	(0.2023)	(0.2084)	(0.2610)
*N*	8873	8873	8873	8873	8873	5476
*R* ^2^	0.6106	0.6101	0.5780	0.5825	0.6023	0.6085

#### 5.4.5. Addressing additional concerns.

First, we address potential omitted variable bias. To avoid bias arising from unobservable factors that vary over time within the industries where private enterprises operate, this study further controls for the interaction fixed effects of “industry × year” (Year × Ind) based on Equation (1). The regression results are shown in column (5) of Table 5. The results indicate that the coefficient of State1 is significantly positive. Second, we exclude the impact of the COVID-19 pandemic. Given that the outbreak from 2020 to 2022 severely disrupted global supply chains and affected firm resilience, we re-estimate the baseline model excluding observations from 2020 to 2022. The regression results are reported in column (6) of Table 5. After excluding the samples during the pandemic period, the impact of state capital participation on the stability of private enterprise supply chains is significantly positive at the 5% level. The above results all indicate that the research conclusions of this study have a certain degree of robustness.

### 5.5. Test of the mechanism of action

The positive and robust relationship between state-owned capital participation and supply chain stability motivates further investigation into the underlying mechanisms. Based on theoretical arguments of this study, two potential channels are proposed: One is alleviating pressure from resource constraints, and the other is mitigating principal-agent conflicts. To test these hypotheses, this study draws on Jiang (2022) [60] regarding the examination of underlying mechanisms and, combined with a two-way fixed effects model, constructs model (4) to test the mediating effects:


$$M_{it}=\mu_{\text{0}}+\mu_{\text{1}}{State\text{1}}_{it}+\mu_{\text{2}}Controls+Year+Firm+\varepsilon_{it}$$ (4)

Where M represents each hypothesized mechanism variable in turn, and the rest remain consistent with the benchmark regression model.

#### 5.5.1. Resource empowerment.

The alleviation of resource constraints can enable private enterprises to make timely payments and fulfill their contractual obligations, thereby strengthening the stability of supply chain relationships. To examine the resource empowerment mechanism, this study assesses the resource constraint pressure faced by private enterprises from two dimensions: financial resources and political resources. For financial resources, the financing constraint index (FC) is employed as its proxy variable, with a higher value indicating more severe financing constraints. For political resources, this study uses the logarithm of government subsidies plus one (Subsidy) as its proxy variable. The regression results are shown in columns (1) and (2) of Table 6. The estimated coefficient of State1 in column (1) is significantly negative, indicating that state-owned capital participation mitigates financing constraints faced by private enterprises. The estimated coefficient of State1 in column (2) is significantly positive, indicating that state-owned capital participation can increase the amount of government subsidies received by private enterprises. Sobel tests further confirm the mediating role of resource empowerment, with Z-statistics for FC and Subsidy being 2.824 and 3.321, respectively, both of which are significantly positive at the 1% level. Collectively, these findings indicate that state-owned capital participation reduces resource constraint pressure and enhances supply chain stability among private enterprises, thus supporting Hypothesis H1a. Considering the results from columns (1) to (2) collectively, it can be inferred that state-owned capital participation can alleviate the resource constraint pressure of private enterprises and thereby enhance their supply chain stability. Hypothesis H1a is verified. To address potential endogeneity between state-owned capital participation and resource empowerment, lagged resource variables are incorporated into the model, and system GMM estimation is applied. The results in Table 6, Panel B, columns (1) and (2), show that the effect of state-owned capital participation on resource empowerment is consistent with Panel A and passes the AR (1), AR (2), and Sargan tests. Therefore, even after considering endogeneity, hypothesis H1a is supported.

**Table 6:** Results of Mechanism of Action Testing.

Panel A				
VARIABLES	Resource Pressure		Principal-agency Conflicts	
	(1)	(2)	(3)	(4)
	*FC*	*Subsidy*	*AC1*	*AC2*
Panel A				
*State1*	−0.3070^***^	1.0410^**^	−0.0499^*^	−0.0277^**^
	(0.0709)	(0.5213)	(0.0296)	(0.0124)
Controls	YES	YES	YES	YES
Firm & Year	YES	YES	YES	YES
Constant	4.306^***^	−3.631^***^	0.450^***^	0.0696^***^
	(0.0811)	(0.5682)	(0.0318)	(0.0138)
*N*	8584	8312	8429	8432
*R* ^2^	0.874	0.803	0.715	0.549
*Sobel*	Z = 2.824^***^	Z = 3.321^***^	Z = 2.147^**^	Z = 3.448^***^
Panel B				
*State1*	−0.0736*** (0.0285)	0.0892** (0.0392)	−0.0924*** (0.0357)	−0.0683** (0.0271)
*L.FC*	0.1723***			
	(0.0516)			
*L.Subsidy*		0.2156***		
		(0.0642)		
*L.AC1*			0.1489***	
			(0.0437)	
*L.AC2*				0.1932***
				(0.0578)
Controls	YES	YES	YES	YES
Firm & Year	YES	YES	YES	YES
Constant	3.1416*** (0.3826)	1.5927*** (0.5832)	−0.8912*** (0.2937)	0.4633*** (0.1285)
N	7442	7442	7442	7442
AR (1) test	P value = 0.023	P value = 0.041	P value = 0.018	P value = 0.037
AR (2) test	P value = 0.317	P value = 0.289	P value = 0.402	P value = 0.358
Sargan test	P value = 0.156	P value = 0.203	P value = 0.115	P value = 0.247

#### 5.5.2. Internal governance structure empowerment.

Reducing principal-agent conflicts can increase the trust of upstream suppliers and downstream customers in private enterprises, thereby enhancing their supply chain stability Private enterprises face two types of principal-agent conflicts. The first type of principal-agent conflict arises between shareholders and managers, where managers, possessing informational advantages, may manipulate corporate information to serve private interests. This paper refers to the research by Ang et al. (2000) and selects the management expense ratio as the proxy variable for the first type of principal-agent conflict (AC1), with a higher value indicating a more severe first type of principal-agent conflict [61]. The second type of principal-agent conflict occurs between majority shareholders and minority shareholders. Given that most private enterprises in China are controlled by an individual or family with highly concentrated ownership, majority shareholders exploit their control to divert corporate resources. Referring to the research by Wang et al. (2022), the ratio of other receivables to total assets is adopted as a proxy for this conflict (AC2), where a higher ratio signifies more severe expropriation behavior [11]. The regression results are shown in columns (3) and (4) of Table 6. In column (3), the estimated coefficient of State1 is significantly negative, indicating that state-owned capital participation significantly reduces the first type of principal-agent conflict in private enterprises. In column (4), the estimated coefficient of State1 is also significantly negative, indicating that state-owned capital participation significantly reduces the second type of principal-agent conflict in private enterprises. Sobel tests yield Z-statistics of 2.901 for both AC1 and AC2, significant at least at the 5% level, providing strong support for the mediating effect. These results offer robust causal evidence for Hypothesis H1b. To mitigate the endogeneity concerns, this study includes lagged internal governance structure empowerment variables in the regression model. A system GMM estimation is then conducted. The results, found in Table 6, Panel B, columns (3) and (4), show that the effect of state-owned capital participation remains consistent with the findings in Panel A and passes the AR (1), AR (2), and Sargan tests, confirming the robustness of H1b even when endogeneity is considered.

## 6. Heterogeneity analysis

The regression results presented earlier provide strong support for the causal relationship between state-owned capital participation and enhanced supply chain stability in private enterprises, validating two underlying mechanisms: improved information disclosure quality and enhanced financial status. However, further discussion is needed to determine whether the impact varies under different contextual factors. In practice, supply chain stability is influenced by inter-organizational, intra-organizational, and external factors. Therefore, drawing on existing literature, this study conducts a heterogeneity analysis along three dimensions: inter-organizational factors (bank-enterprise relationships), intra-organizational factors (product advantages, internal control levels), and external factors (media coverage), aiming to offer more nuanced and actionable insights for policy design and managerial practice.

### 6.1. Heterogeneity analysis based on bank-enterprise relationships (From the Perspective of Inter-Organizational Relationships)

From the standpoint of bank-enterprise relationships, firms with such ties can typically secure higher credit limits [20], thereby alleviating the financing constraints and improving their financial status. Consequently, strong bank-enterprise relationships may partially substitute for the financial benefits brought by state-owned capital participation, weakening its incremental impact on financial improvement. Therefore, we hypothesize that the positive effect of state-owned capital participation on supply chain stability is more pronounced for private enterprises lacking bank-enterprise relationships. To test this hypothesis, this study follows the approach of existing research [62] and divides the research sample into two groups: those with and without bank-enterprise relationships. A firm is classified as having a bank-enterprise relationship if it meets any of the following criteria: bank-background executives, banks holding shares in the enterprise, or the enterprise holding shares in a bank, otherwise, it is considered not to have such a relationship. The regression results, reported in columns (1) and (2) of Table 7, indicate that the estimated coefficient of State1 is not significant in the sample of private enterprises with bank-enterprise relationships, but it is significantly positive in the sample of private enterprises without bank-enterprise relationships. This suggests that, the effect of state-owned capital participation on enhancing supply chain stability is stronger for private enterprises without established bank ties.

**Table 7:** Results of Heterogeneity Tests: Bank-enterprise Relationship Grouping and Product Advantage Grouping.

VARIABLES	Bank-Enterprise Relationship Grouping		Product Advantage Grouping	
	Good	Bad	Strong	Weak
	(1)	(2)	(3)	(4)
	*Stability*	*Stability*	*Stability*	*Stability*
*State1*	0.5839	0.5305^**^	0.4505	0.7732^***^
	(0.3582)	(0.2077)	(0.2857)	(0.2313)
Controls	YES	YES	YES	YES
Year & Firm	YES	YES	YES	YES
Constant	0.0136	0.5596^**^	−0.1882	0.6897^**^
	(0.3623)	(0.2401)	(0.2896)	(0.2905)
*N*	2566	6307	4390	4483
*R* ^2^	0.6562	0.6645	0.6703	0.6831

### 6.2. Heterogeneity analysis based on product advantages (From the Intra-Organizational Perspective)

Product advantages determine an enterprise’s bargaining power and strategic position in transactions. Upstream suppliers are more inclined to allocate resources to firms with strong product advantages, as they benefit from association with reputable brands. Downstream customers seek technologically advanced and higher-quality inputs to strengthen their own competitiveness and expand market share. Therefore, compared to private enterprises with higher product advantages, those with lower product advantages are less preferred as partners by upstream and downstream enterprises, making it harder for them to maintain stable supply chain partnerships. As a result, this study infers that state-owned capital participation increases supply chain stability in private enterprises, with a more pronounced effect for those with lower product advantages. To examine this, this study divides the research sample into two groups using the industry annual median of main business revenue growth rate: private enterprises with higher product advantages and those with lower product advantages. The groups are then estimated separately. Regression results in columns (3) and (4) of Table 7 show that the coefficient of State1 is significantly positive for private enterprises with lower product advantages but not significant for those with higher product advantages. This supports the view that the stabilizing effect of state-owned capital participation is more evident in private enterprises with lower product advantages.

### 6.3. Heterogeneity analysis based on internal control levels (From the Intra-Organizational Perspective)

Internal control quality reflects corporate governance and plays a critical role in determining agency costs within firms [63]. A well-functioning internal control system curbs opportunistic behavior of controlling shareholders and management. It also reduces the likelihood of concealing adverse information and enhances the quality of information disclosure [64]. Therefore, strong internal control can attenuate the governance-enhancing effect of state-owned capital participation in private enterprises. Based on this reasoning, we hypothesize those private enterprises with weaker internal control experience a more pronounced improvement in supply chain stability following state-owned equity participation, compared to those with stronger internal control. To test hypothesis, we use the internal control index published by DiBo Company as a measure of internal control level. The research sample is divided into two groups based on the annual median: enterprises with better internal control and those with poorer internal control systems. Grouped testing is conducted. The regression results presented in columns (1) and (2) of Table 8 show that the coefficient on State1 is significantly positive for the group with poorer internal control, but insignificant for the group with better internal control. This suggests that the positive effect of state-owned capital participation on supply chain stability is more pronounced among private enterprises with weaker internal governance systems.

**Table 8:** Results of Heterogeneity Tests: Internal Control Group and Media Negative Reporting Group.

VARIABLES	Internal Control Group		Media Negative Reporting Group	
	High	Low	Many	Few
	(5)	(6)	(7)	(8)
	*Stability*	*Stability*	*Stability*	*Stability*
*State1*	0.4361	0.6873^***^	0.5208^**^	0.4386
	(0.3018)	(0.2259)	(0.2283)	(0.2841)
Controls	YES	YES	YES	YES
Year & Firm	YES	YES	YES	YES
Constant	0.2809	0.4089	0.5672^**^	0.0720
	(0.3147)	(0.2634)	(0.2557)	(0.3077)
*N*	4205	4668	4539	4334
*R* ^2^	0.6816	0.6638	0.6716	0.6616

### 6.4. Heterogeneity analysis based on negative media coverage (From the Extra-Organizational Perspective)

Media coverage serves as a crucial channel through which external stakeholders obtain corporate information. Compared to positive media reports, negative media coverage attracts more attention from stakeholders, including upstream suppliers and downstream customers [65]. Once adverse information such as poor financial performance appears in negative reports, it can trigger pessimistic expectations among supply chain partners, thereby undermining the stability of corporate supply chains. Considering this, we hypothesize that compared to private enterprises with fewer negative media reports, the positive effect of state-owned capital participation on the supply chain stability of private enterprises is more pronounced in those with more negative media reports. To verify this theoretical inference, the sample is split into two groups based on the annual median number of negative online media reports about each firm, and subgroup analyses are performed. The regression results in columns (3) and (4) of Table 8 indicate that estimated coefficient of State1 is insignificant in the low-negative-media group but significantly positive in the high-negative-media group. This suggests that compared to private enterprises with fewer negative media reports, the positive effect of state-owned capital participation on the supply chain stability of private enterprises is more evident in those with more negative media reports, thereby confirming the theoretical inference.

However, recognizing the potential limitations and biases that may arise from using grouping variables for heterogeneity, The interactive fixed effects in the model were controlled. The results are shown in Table 9. As shown in Table 9, incorporating the interactive fixed effects (Year × Ind) does not alter the conclusions of this paper.

**Table 9:** Heterogeneity Test Results (with interactive fixed effects included).

VARIABLES	Bank-Enterprise Relationship Grouping		Product Advantage Grouping		Internal Control Group		Media Negative Reporting Group	
	Good	Bad	Strong	Weak	High	Low	Many	Few
	(1)	(2)	(3)	(4)	(5)	(6)	(7)	(8)
	*Stability*	*Stability*	*Stability*	*Stability*	*Stability*	*Stability*	*Stability*	*Stability*
*State1*	0.4578	0.8122^***^	0.2233	0.6405^***^	0.1374	0.5125^**^	0.5903^*^	0.3687
	(0.4237)	(0.1235)	(0.3157)	(0.2537)	(0.3267)	(0.2476)	(0.2283)	(0.2462)
Controls	YES	YES	YES	YES	YES	YES	YES	YES
Year & Firm	YES	YES	YES	YES	YES	YES	YES	YES
Year × Ind	YES	YES	YES	YES	YES	YES	YES	YES
Constant	1.4826^***^	−4476^***^	−0.1882	0.6897^**^	0.2809	0.4089	0.4142	1.2751
	(0.5095)	(0.1134)	(0.2896)	(0.2905)	(0.3147)	(0.2634)	(0.3901)	(0.3336)
*N*	2566	6307	4390	4483	4205	4668	4539	4334
*R* ^2^	0.6738	0.2243	0.6549	0.6810	0.6637	0.6659	0.6897	0.6566

## 7. Conclusions and implications

### 7.1. Conclusions

Using data from A-share listed private enterprises from 2013 to 2022 and drawing on the empowerment theory, this study empirically examines the impact of state capital participation on the stability of private enterprise supply chains. The following conclusions are drawn: First, we develop a theoretical framework explaining how state capital involvement enhances supply chain stability, providing a theoretical reference for developing countries to introduce state capital and improve supply chain stability when private enterprises experience fluctuations in their supply chain stability. Second, state capital participation can increase the stability of private enterprise supply chains. This conclusion remains robust across a series of tests, including the instrumental variable method, matching method, difference-in-differences method, alternative variable measures, subsample regression, and interaction fixed effects. Third, the mechanism test reveals that the improvement stems from the alleviation of resource constraints and principal-agent conflicts within private enterprises. Finally, for those private enterprises that lack bank partnerships, have weak product advantages, poorer internal control, and more negative media coverage, the positive impact of government capital participation on the stability of the private enterprise supply chain is more significant. This has policy implications for optimizing the allocation of state capital.

### 7.2. Implications

The findings of this study carry important policy implications and practical value in deepening the reverse mixed-ownership reform and strengthening the resilience of private enterprise supply chains. For policymakers, given the significant positive effect of state-owned capital participation on the supply chain stability of private enterprises, it is advisable to further refine the implementation framework of reverse mixed-ownership reform. On the one hand, differentiated mechanisms for state-owned capital involvement should be established. Targeted support policies can be introduced for private enterprises facing weak banking relationships, limited product competitiveness, inadequate internal controls, or predominantly negative media coverage. Through measures such as tax incentives and simplified approval procedures, capital state-owned capital can be directed more precisely to areas of greatest need, thereby alleviating resource constraints and promoting governance upgrades. For example, private enterprises encountering financing difficulties can be encouraged to access state-owned capital via equity financing, supported by resource-matching platforms that facilitate connections with critical upstream and downstream supply chain partners. On the other hand, a dynamic regulatory system for state-owned capital participation should be constructed. While leveraging its capacity to enhance resources and optimize governance, reasonable limits on shareholding ratios and well-defined boundaries for administrative intervention must be set to prevent excessive interference. This ensures that improvements in supply chain stability arise from enhanced operational efficiency rather than external coercion. For private enterprises, it is crucial to proactively seize opportunities arising from the reverse mixed-ownership reform and formulate state-owned capital introduction strategies tailored to their own operational weaknesses. Firms with limited resource acquisition capabilities can establish long-term, stable resource supply channels by introducing state-owned capital and leveraging its credit endorsement to strengthen collaboration with suppliers and customers. Enterprises with internal governance deficiencies can utilize the participation of state-owned capital to enhance corporate charters, refine board structures, and mitigate the adverse effects of principal-agent conflicts on supply chain cooperation. For instance, establishing regular communication mechanisms between state-owned shareholder representatives and management can enhance decision-making transparency and execution efficiency. Additionally, industry associations can serve as intermediaries by identifying common supply chain pain points of private enterprises, building matchmaking platforms to connect state-owned capital with private firms, and organizing training programs on governance optimization. Such initiatives can facilitate the translation of research findings into practice, enabling private enterprises to build more resilient supply chains in complex market environments and supporting the sustained and healthy development of the private economy.

This study has several limitations. First, it relies on data from China’s Shanghai and Shenzhen A-shares to examine the impact of state capital participation on the stability of private enterprise supply chains. However, by focusing solely on listed companies, many non-listed private enterprises are overlooked. These enterprises are often concentrated in sectors such as small-scale manufacturing and traditional services, which are integral parts of the national economy. Therefore, the research results may not capture how state-owned capital participation influences supply chain stability in these industries, potentially leading to biased assessments of its role of state capital participation and an incomplete understanding of different industry characteristics on the relationship between state capital participation and supply chain stability. Due to constraints in data accessibility, we are currently unable to conduct research using samples of non-listed enterprises. We hope to address this limitation by incorporating broader enterprise populations in future work.

## Supporting information

S1 Table Data. (XLSX)

## References

[1] Huang K, Liu G, Chen B, Zhang Z. From Relational Stability to Network Resilience: Annual Negotiated versus Competitive Municipal Bond-Issuing Networks. Am Rev Public Administ. 2025;55(6):509–27. doi: 10.1177/02750740251346808

[2] Gu J, Shi X, Wang P, Xu X. Examining the impact of upstream and downstream relationship stability and concentration on firms’ financial performance. J Bus Res. 2022;141:229–42. doi: 10.1016/j.jbusres.2021.12.018

[3] Geng Y, Wang Y, Wu S. Acquaintance means booster? Why stable customers matter for firm productivity. China J Account Res. 2025;18(3):100435. doi: 10.1016/j.cjar.2025.100435

[4] Daganzo CF. On the Stability of Supply Chains. Operat Res. 2004;52(6):909–21. doi: 10.1287/opre.1040.0147

[5] Abimanyu Y, Rokhim R. The Effect Of State Capital Participation On The Financial And Non-Financial Performance Of State-Owned Enterprises And Other Institutions. Cakrawala Repositori IMWI. 2023;6(6):2539–57. doi: 10.52851/cakrawala.v6i6.556

[6] Xu S, Chen S, Jiao W, Chen M. State-owned enterprises shareholders and innovation of private enterprises: Evidence from China. Technovation. 2025;140:103144. doi: 10.1016/j.technovation.2024.103144

[7] Xue H, Zhang H, Zhang X, Ding S. The role of state-owned capital in the innovation of private-owned enterprises: Evidence from China. Pacific-Basin Financ J. 2026;96:103031. doi: 10.1016/j.pacfin.2025.103031

[8] Xiao L, Ge C, Luo Z, Zhang W, Chen Z. How partial nationalizations affect technological innovation in mixed-ownership enterprises: A theoretical explanation based on the effects of heterogeneous shareholder governance and resource acquisition. Int Rev Econ Financ. 2024;94:103404. doi: 10.1016/j.iref.2024.103404

[9] Zhao Y, Mao J. Mixed ownership reforms and the transparency of nonstate‐owned enterprises: Evidence from China. Manage Decis Econ. 2022;44(1):271–84. doi: 10.1002/mde.3679

[10] Li S, Yu X, Qian K. How does the state capital participation optimize the corporate green innovation structure: Evidence from listed private firms. Int Rev Financ Analys. 2024;96:103671. doi: 10.1016/j.irfa.2024.103671

[11] Li P, Yuan X, Jin L. Government direct intervention and stock market concentration. Appl Econ. 2022;55(25):2863–74. doi: 10.1080/00036846.2022.2107609

[12] Yang X, Zhang K, Gao P, Yang Z. State-owned shareholders’ participation and environmental, social, and governance performance of private firms: evidence from China. Appl Econ. 2025;57(26):3503–24. doi: 10.1080/00036846.2024.2337797

[13] Chen C. Access to finance: How does state share participation reduce management costs in private firms? Int Rev Econ Financ. 2025;104:104699. doi: 10.1016/j.iref.2025.104699

[14] Chen L, Gao F, Guo T, Huang X. Mixed ownership reform and the short-term debt for long-term investment of non-state-owned enterprises: Evidence from China. Int Rev Financ Analys. 2023;90:102861. doi: 10.1016/j.irfa.2023.102861

[15] Yang Y, Qian Y, Li S. State capital and cash holdings in natural private enterprises: New evidence and a new explanation. Financ Res Lett. 2023;51:103393. doi: 10.1016/j.frl.2022.103393

[16] Tang Y, Yang T, Chen J, Li Z. State ownership and green innovation in family firms. Int Rev Financ. 2025;25(3). doi: 10.1111/irfi.70039

[17] Zhang R, Lin Y, Zhang W, Du J. Mixed ownership reform of state-owned enterprises and executive compensation stickiness: Evidence from China. Int Rev Econ Financ. 2024;95:103432. doi: 10.1016/j.iref.2024.103432

[18] Yao M, Song C, Song Z. State ownership, political connections and entry barriers: evidence from China. Appl Econ Lett. 2018;25(17):1250–4. doi: 10.1080/13504851.2017.1414928

[19] Chen H, Rithmire M. The Rise of the Investor State: State Capital in the Chinese Economy. St Comp Int Dev. 2020;55(3):257–77. doi: 10.1007/s12116-020-09308-3

[20] Jin X, Yu J, Yuan G, Zang R. Impact of State‐Owned Equity Participation on the Risk‐Taking Capacity of Private Enterprises in China: Insights From a Quasinatural Experiment. Corpor Govern. 2024;33(4):629–62. doi: 10.1111/corg.12619

[21] Xiao Z, Niu Q, Yun F, Ye Y. The impact of state-owned capital on labor cost stickiness in private firms: Evidence from China. Econ Modell. 2024;141:106906. doi: 10.1016/j.econmod.2024.106906

[22] Hu C, Li Y, Ye P. The Halo Effect of Government: Does State-Owned Capital Promote the Green Innovation of Chinese Private Enterprises? Sustainability. 2023;15(11):8587. doi: 10.3390/su15118587

[23] Ye Y, Zhang L. Mixed ownership reform helps stabilize employment? Discussion on the entry of state-owned capital and the employment absorption capacity of private enterprises. Indust Econ Res. 2022;(02):57–70. doi: 10.13269/j.cnki.ier.2022.02.003

[24] Zhang T, Gu L, Wang JJ. State‐owned capital and corporate social responsibility of private‐holding companies: evidence from China. Account Financ. 2022;63(S1):1101–20. doi: 10.1111/acfi.12931

[25] Guo M, Li N, Guo F, Li X. The State Capital Investing and Operating Company Pilot Reform and the Financialization of the Chinese SOEs. Financ Manag Account. 2025;37(1):63–79. doi: 10.1111/jifm.12241

[26] Chen C, Qian J, Zhu X, Leng X, Hao C, Xu B. Can state-owned capital investment improve the financial performance of Chinese private enterprises? Appl Econ. 2025;:1–14. doi: 10.1080/00036846.2025.2492340

[27] Yao Y, Huaijin Q. Mixed ownership and healthy development of private economy: Based on the perspective of enterprise violations. J Financ Econ. 2022;48(03):33–47. doi: 10.16538/j.cnki.jfe.20211218.302

[28] Dong H, Yan Q. Can supply chain stability achieve stable employment? J Financ Econ. 2024;51(02):123–37. doi: 10.16538/j.cnki.jfe.20240118.103

[29] Tu Y, Hu L, Hua X, Li H. Supply chain stability and corporate green technology innovation. Int Rev Econ Financ. 2025;97:103769. doi: 10.1016/j.iref.2024.103769

[30] Patatoukas PN. Customer-base concentration: Implications for firm performance and capital markets: 2011 American accounting association competitive manuscript award winner. Account Rev. 2012;87(2):363–92. doi: 10.2308/accr-10198

[31] Hendricks KB, Singhal VR. Association Between Supply Chain Glitches and Operating Performance. Manag Sci. 2005;51(5):695–711. doi: 10.1287/mnsc.1040.0353

[32] Ma J, Gao D. The Impact of Sustainable Supply-Chain Partnership on Bank Loans: Evidence from Chinese-Listed Firms. Sustainability. 2023;15(6):4843. doi: 10.3390/su15064843

[33] Jia F, et al. Does supply chain concentration improve sustainability performance: the role of operational slack and information transparency. Int J Operat Product Manag. 2025;45(1):269–300. doi: 10.1108/IJOPM-12-2022-0807

[34] Zhang S, Gu C. Supply chain digitalization and supply chain resilience. J Financ Econ. 2024;50(07):21–34. doi: 10.16538/j.cnki.jfe.20231017.101

[35] Jin JL, Wang L, Wang K, Fu X. Concentrating or dispersing? The double-edged sword effects of supplier concentration on firm financial and innovation performance. J Bus Res. 2025;186:114946. doi: 10.1016/j.jbusres.2024.114946

[36] Baiman S, Rajan MV. The Role of Information and Opportunism in the Choice of Buyer‐Supplier Relationships. J Account Res. 2002;40(2):247–78. doi: 10.1111/1475-679x.00046

[37] Bauer AM, Henderson D, Lynch DP. Supplier Internal Control Quality and the Duration of Customer-Supplier Relationships. Account Rev. 2017;93(3):59–82. doi: 10.2308/accr-51889

[38] Chen X, Wan P, Wang Q. Does ESG decoupling reduce customer stability? Evidence from China. Appl Econ. 2024;57(42):6593–611. doi: 10.1080/00036846.2024.2385756

[39] Jin H. Challenges to corporate supply chain stability under the trend of expert power concentration. Int Rev Financ Analys. 2025;97:103820. doi: 10.1016/j.irfa.2024.103820

[40] Monje-Amor A, Xanthopoulou D, Calvo N, Abeal Vázquez JP. Structural empowerment, psychological empowerment, and work engagement: A cross-country study. Eur Manag J. 2021;39(6):779–89. doi: 10.1016/j.emj.2021.01.005

[41] Hsieh SH, Lee CT, Tseng TH. Psychological empowerment and user satisfaction: Investigating the influences of online brand community participation. Inform Manag. 2022;59(1):103570. doi: 10.1016/j.im.2021.103570

[42] Barney J. Firm Resources and Sustained Competitive Advantage. J Manag. 1991;17(1):99–120. doi: 10.1177/014920639101700108

[43] He D, Zeng M, Zhang S. How do state-owned capital shareholders affect private enterprises? Research from the perspective of debt financing. J Manag World. 2022;38(11):189–207. doi: 10.19744/j.cnki.11-1235/f.2022.0160

[44] Wei C, Wei X, Can Z. Resignation of independent directors, restructuring of political ties, and the introduction of state-owned capital in private enterprises: Evidence from China. International Review of Financial Analysis. 2025;105:104406. doi: 10.1016/j.irfa.2025.104406

[45] Li X, Xu Q, Guo F, Wang H. State‐owned equity participation and private sector enterprises’ strategic risk taking: Evidence from China. Manage Decis Econ. 2022;44(2):1107–24. doi: 10.1002/mde.3735

[46] Ghadge A, Jena SK, Kamble S, Misra D, Tiwari MK. Impact of financial risk on supply chains: a manufacturer-supplier relational perspective. Int J Product Res. 2020;59(23):7090–105. doi: 10.1080/00207543.2020.1834638

[47] Lin D. Accelerability vs. scalability: R&D investment under financial constraints and competition. Manag Sci. 2023;69(7):4078–107. doi: 10.1287/mnsc.2022.4503

[48] Acebo E, Miguel-Dávila J-Á, Nieto M. Do financial constraints moderate the relationship between innovation subsidies and firms’ R&D investment? EJIM. 2020;25(2):347–64. doi: 10.1108/ejim-07-2020-0286

[49] Liu Y, Du J, Kang T, Kang M. Establishing supply chain transparency and its impact on supply chain risk management and resilience. Oper Manag Res. 2024. doi: 10.1007/s12063-024-00499-9

[50] Jensen MC, Meckling WH. Theory of the firm: Managerial behavior, agency costs and ownership structure. J Financ Econ. 1976;3(4):305–60. doi: 10.1016/0304-405x(76)90026-x

[51] Toninelli PA. The rise and fall of state-owned enterprise in the Western world. Cambridge University Press; 2000. p. 49–72.

[52] Peng X, Jia Y, Chan KC. The impact of internationalization on IPO underpricing: A result of agency costs reduction, a certification effect, or a diversification benefit? Finan Res Lett. 2022;44:102059. doi: 10.1016/j.frl.2021.102059

[53] Huang S, Du P, Hong Y. Can state-owned equity participation improve a company’s environmental, social and governance performance? Evidence collected from China. SAMPJ. 2024;16(1):226–58. doi: 10.1108/sampj-05-2023-0284

[54] Zhu R, Xin X, Tan K. Reverse mixed ownership reform: Does state-owned capital injection inhibit corporate leverage manipulation? Finan Res Lett. 2024;59:104764. doi: 10.1016/j.frl.2023.104764

[55] Garen JE. Executive Compensation and Principal-Agent Theory. J Polit Econ. 1994;102(6):1175–99. doi: 10.1086/261967

[56] Dsouza M. The corporate agent in criminal law – an argument for comprehensive identification. CLJ. 2020;79(1):91–119. doi: 10.1017/s0008197320000021

[57] Lotfi Z, et al. Information sharing in supply chain management. Procedia Technol. 2013;11:298–304. doi: 10.1016/j.protcy.2013.12.194

[58] Aktas N, Andreou PC, Karasamani I, Philip D. CEO Duality, Agency Costs, and Internal Capital Allocation Efficiency. British J Manag. 2019;30(2):473–93. doi: 10.1111/1467-8551.12277

[59] Chen Z, Cao Y, Liao K. How state-owned equity participation promotes the digital transformation of nonstate-owned enterprises: Evidence from China. Finan Res Lett. 2024;59:104818. doi: 10.1016/j.frl.2023.104818

[60] Jiang T. Mediating Effects and Moderating Effects in Causal Inference. China Indust Econ. 2022(05):100–20. doi: 10.19581/j.cnki.ciejournal.2022.05.005

[61] Ang JS, Cole RA, Lin JW. Agency Costs and Ownership Structure. J Finan. 2000;55(1):81–106. doi: 10.1111/0022-1082.00201

[62] Shao L. Impacts of bank enterprise relationship on debt financing and enterprise investment efficiency. Res Financ Econ Issues. 2018;(09):76–82. doi: 10.19654/j.cnki.cjwtyj.2018.09.009

[63] Doyle J, Ge W, McVay S. Determinants of weaknesses in internal control over financial reporting. J Account Econ. 2007;44(1–2):193–223. doi: 10.1016/j.jacceco.2006.10.003

[64] Caplan D. Internal Controls and the Detection of Management Fraud. J Account Res. 1999;37(1):101. doi: 10.2307/2491398

[65] Wang L, Ye Y, Huo B. Spillover of bad publicity: Effect of negative ESG coverage in supply chains on firm performance. Int J Product Econ. 2025;285:109654. doi: 10.1016/j.ijpe.2025.109654

